# *Streptococcus equi* subspecies *zooepidemicus* – a case report of sudden death in a German sow farm

**DOI:** 10.1186/s40813-023-00344-8

**Published:** 2023-10-24

**Authors:** Lukas Geiping, Henrik Detlefsen, Sara Trittmacher, Christoph Georg Baums, René Bergmann, Isabel Hennig-Pauka

**Affiliations:** 1 Field Station for Epidemiology in Bakum, University of Veterinary Medicine Hannover, Foundation, Germany; 2 Tierarztpraxis Bethen, Heideweg 7, 49661 Cloppenburg/Bethen, Germany; 3 Institute of Bacteriology and Mycology, Centre for Infectious Diseases, Faculty of Veterinary Medicine, Leipzig University, Leipzig, Germany

**Keywords:** Swine, Septicaemia, Mortality, Bacteriaemia, Neutrophilia, Sequence typing

## Abstract

A farm in North-West Germany experienced a high morbidity and mortality in their sow herd. Sows showed fever, lethargy, oedema, mucosal discharge and dyspnoea. Necropsy revealed a severe fibrinous and purulent polyserositis. Haematological and histological examinations confirmed septicaemia. *Streptococcus equi subspecies zooepidemicus* was isolated in high yields from major organs. Sequence typing of this isolate (21/455) revealed a new sequence type showing a significantly higher proliferation rate in comparison to two other isolates. Other infectious agents (influenza A virus, Porcine Reproductive and Respiratory Syndrome Virus, Porcine Circovirus 2, african swine fever virus, classical swine fever virus, *Actinobacillus pleuropneumoniae*) were excluded by routine diagnostic examinations. A climate check revealed an insufficient air supply in the area for the gestating sows. This case describes the first disease outbreak in swine due to *S. zooepidemicus* in Germany.

## Background

*Streptococcus equi* subspecies *zooepidemicus* (*S. zooepidemicus*) is a Gram-positive, coccoid, beta-haemolytic, Lancefield group C bacterium. This commensal and opportunistic pathogen in warm-blooded hosts, including humans [1] is a major pathogen in horses and is associated with different diseases such as abortion, arthritis in foals, pneumonia, septicaemia, and meningitis. *S. zooepidemicus* can also be involved in respiratory diseases in dogs [2]. Sudden death and respiratory disease in pigs caused by virulent strains of *S. zooepidemicus* were reported in Canada and the USA [3]. A severe disease outbreak occurred in Sichuan in China in 1975, resulting in 300,000 dead pigs [4–7]. In the following years, *S. zooepidemicus* emerged as a major pathogen in swine in China [8]. Strains isolated in recent outbreaks in North America showed a high homology among each other and to the outbreak strain from 1975 [7]. In clinical trials pigs showed a high morbidity when challenged with strains with a former high pathogenicity but a less susceptibility when infected with horse-associated strains of *S. zooepidemicus* [9, 10]. So far, there is no commercial vaccine available, but various antigens have been tested in experimental studies [8, 11]. Recently, an autogenous vaccine against *S. zooepidemicus* was implemented after a disease outbreak in the Netherlands [12]. In general, implementation of preventive measures against *S. zooepidemicus* is not common in European or American countries. Several virulence factors of *S. zooepidemicus* have been characterized, such as the capsule consisting of hyaluronic acid, the capability of forming biofilms, the M-like protein SzM binding host proteins such as fibrinogen and IgG, a fibronectin-binding protein (FNZ) and the IgG (immunoglobulin G) degrading enzyme of *S. zooepidemicus* (IdeZ) [13–18]. Expression of the hyaluronic acid capsule and SzM is critical for survival and proliferation of *S. zooepidemicus* in blood [16].

## Case presentation

### Farm description

The farrow-to-finishing farm in the North-Western part of Germany close to the Dutch border kept 320 sows in ten groups. It performed all-in-all-out in the farrowing, weaning and fattening stages, with two-week farrowing intervals and a suckling period of 21 days. In total the farm had six farrowing units and two gestating units – a larger for 100–130 sows, the other for 50–70 sows. The smaller unit was located in a separate building together with the breeding centre. Both compartments shared one ventilation-system, while the large gestating unit and the six farrowing units were all equipped with separate ventilation systems. All sows received a commercial diet and water *ad libitum* from a farm-owned well.

Sows were vaccinated routinely against influenza A Virus H3N2, H1N1 and H1N2 every four months. Vaccination against the Porcine Reproductive and Respiratory Syndrome Virus (PRRSV) was performed in every reproduction cycle of a sow group on day 6 after farrowing and on day 60 of gestation (6/60 scheme) using a PRRSV-1 modified-live vaccine. Sows were vaccinated against porcine parvovirus and *Erysipelothrix rhusiopathiae* in the second week of lactation. Piglets were regularly vaccinated against Porcine Circovirus type 2 (PCV2), *Mycoplasma hyopneumoniae* and a PRRSV-1 modified-live vaccine within the first three weeks of life.

Gilts were purchased from a gilt rearing farm 50 km away from the case farm. The gilt rearing farm was under the care of the same veterinarian and had a high health status. Negative results for *Actinobacillus* (*A.*) *pleuropneumoniae* and PRRSV were recorded continuously during routine monitoring. Gilts were introduced to the herd after a six weeks quarantine period in a separate building with outdoor access between a horse holding site and the sow farm. The horse holding site with three horses was about 100 m away from the sow units.

### Case history

The farmer reported fever, lethargy and lack of appetite in his sow herd. Within one day two sudden deaths in gestating sows and one abortion occurred in different compartments of the farm.

Blood samples were taken by the veterinarian from twenty sows showing fever and lethargy. Sows were tested negative for PRRSV and PCV2 by PCR in a routine diagnostic laboratory. Sows were positive for antibodies against influenza A virus in a haemagglutination inhibition assay (HIA), which was difficult to interpret because all sows had been vaccinated. Nasal swabs from suckling piglets and ten diseased sows were tested negative for influenza A virus by PCR. All sows were treated orally with metamizole (Metapyrin^®^ oral 100%, Serumwerk Bernburg, Bernburg, Germany) for five days (50 mg metamizole per kg body weight) and all sows except those in the large gestating unit recovered. In the large gestating unit four sows died in day 102 to 114 of gestation four days after the first clinical signs had been recognized on the farm. Individual sows in this location showed dyspnoea, mucous nasal discharge and oedema of the ears, conjunctiva and nasal bridge. Sows were treated orally with doxycycline (Pulmodox^®^, Virbac, Bad Oldesloe, Germany) in a dosage of 40 mg/kg body weight for five days. Eight days after the first clinical signs had occurred, one sow died and several sows still showed signs of severe disease. The dead sow (sow 454) and one living sow showing signs of severe disease (sow 455) were brought to the Field Station for Epidemiology, Bakum, Germany, for necropsy and subsequent diagnostic measures. The stable climate was checked by a technician and drinking water pipes were sampled at five different locations for bacteriological examination resulting in detection of *Staphylococcus* spp. and yeasts. Six months before, drinking water quality had been checked by standard routine diagnostic procedures resulting in no abnormalities with respect to chemical and microbiological parameters. The inside surfaces of feed silos were checked for dirt and mould, without positive results.

No signs of disease occurred in suckling or nursery piglets, although they were located at the same site as the sows. Fattening pigs were kept 300 m away from the sow site and stayed also healthy. Slaughterhouse data remained constant during the course of the year. On average mild enzootic-pneumonia-like lung lesions in the cranial lobes in 20% of the pigs and mild pleuritis and pericarditis in 5% of the pigs were recorded.

### Pathological findings

**Fig. 1:**
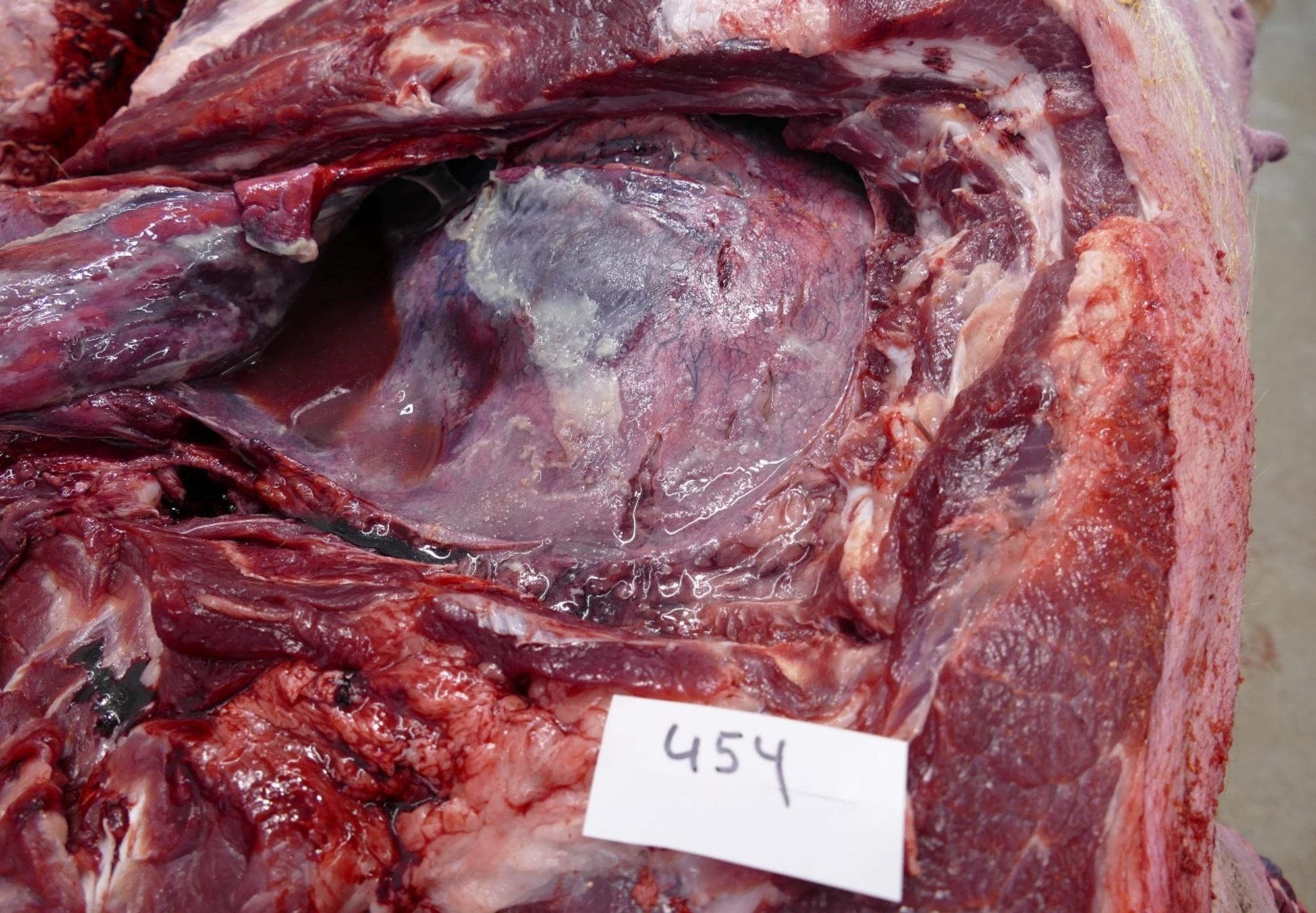
Fibrinous exudate on pleura and pericardium

**Fig. 2:**
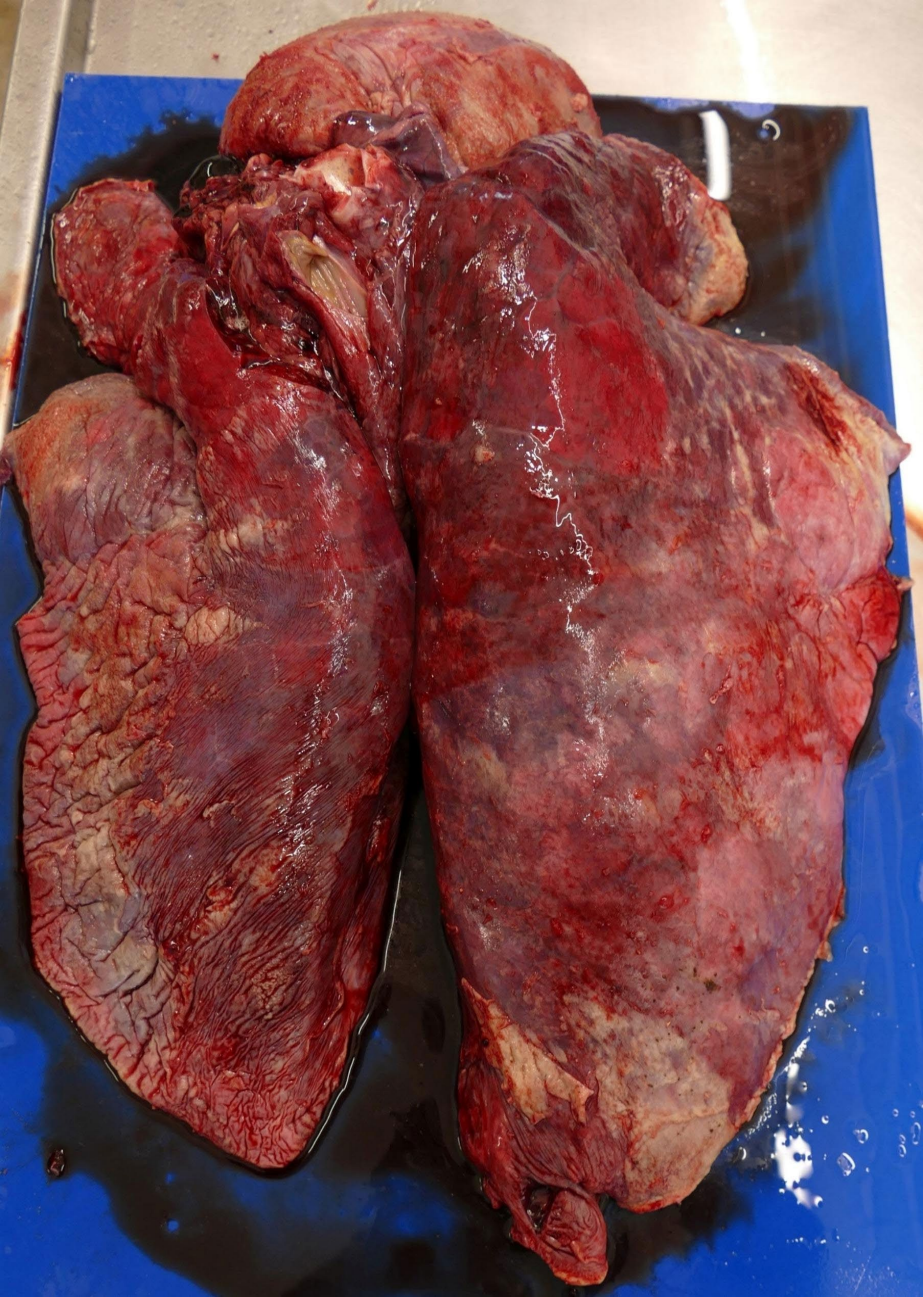
Hyperaemia of the lung and pleuritis

**Fig. 3:**
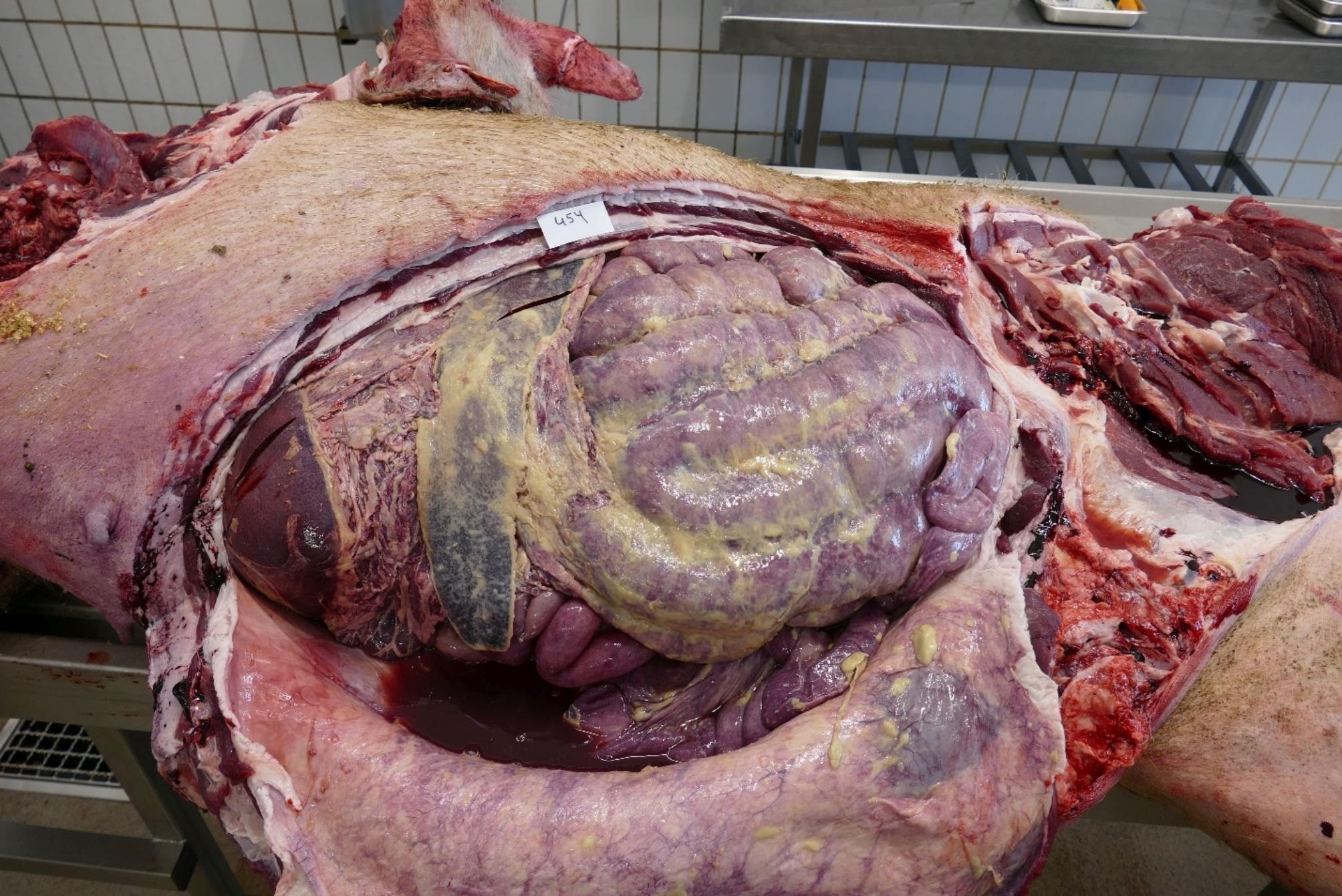
Massive fibrinous exudate in the peritoneal cavity

Post-mortem examination of the dead sow (454) resulted in a severe fibrinous and purulent polyserositis (pleurisy, pericarditis and peritonitis) with a diffuse, fibrinous to serosanguinous exudate in the body cavities and thickening of the serosa (Figs. 1, 2 and 3). Spleen and tracheobronchial lymph nodes were hyperplastic. The lung showed a severe, diffuse haemorrhagic congestion. Trachea and nasal cavities were filled with mucous exudate. Signs of inflammation (radiate, black stripes) were detected in the kidney cortex.

**Fig. 4:**
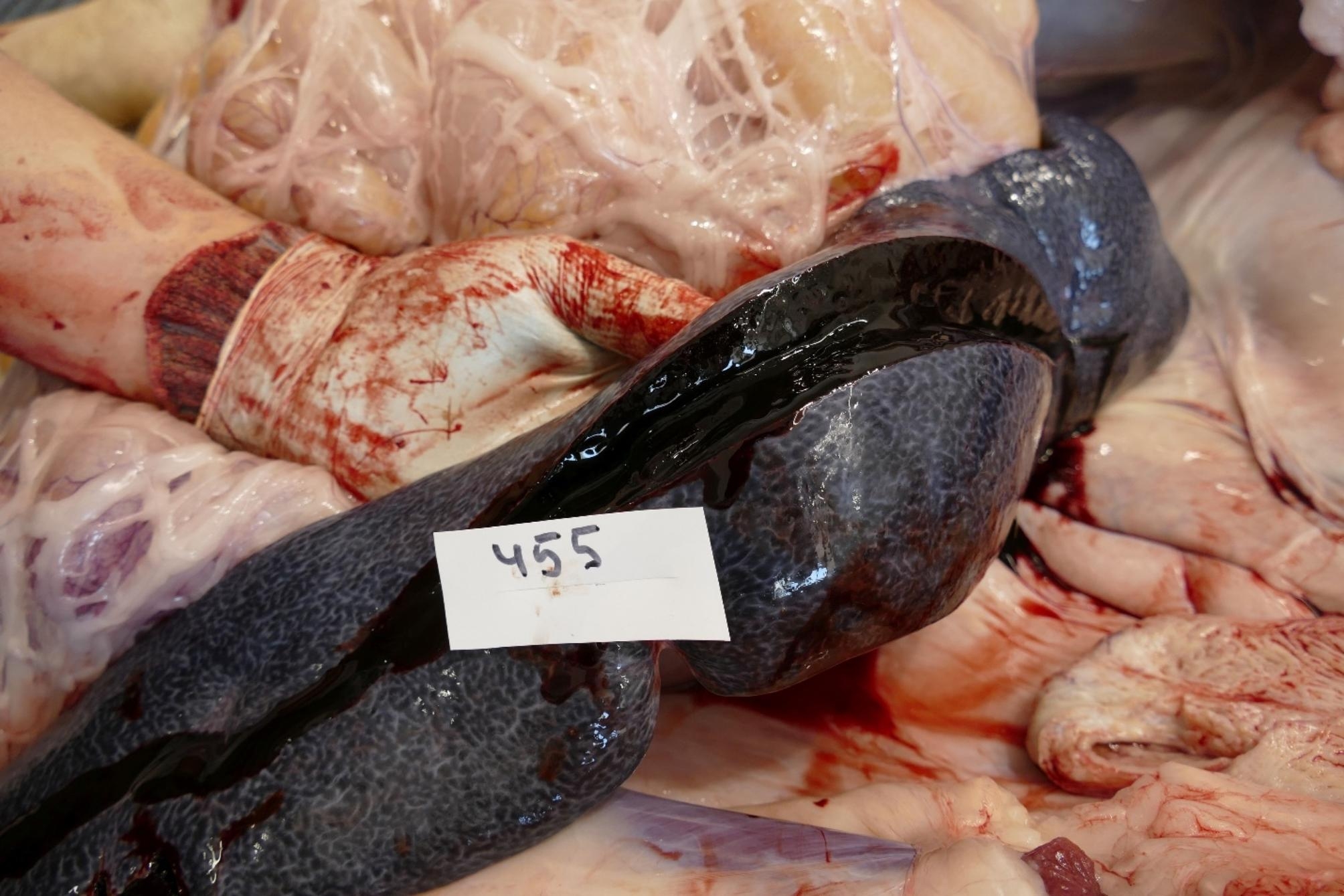
Massive spleen hyperplasia

Sow 455 was euthanized and showed a severe oedema of the ears, a severe congestion of the spleen (Fig. 4) and a severe purulent sinusitis.

### Haematological findings

An analysis of a blood sample from sow 455 resulted in leucocytosis with relative neutrophilia with an increase of juvenile neutrophils, relative lymphopenia and absolute erythropenia (Table 1) pointing to a bacterial infection.

**Table 1:** Blood cell counts

Parameter (unit)	Blood cell counts	Reference ranges
Leukocytes (1000/µl)	30.4	10.12–22.24
Erythrocytes (Mill/µl)	4.15	4.98–7.50
Haemoglobin (g/dl)	8.9	10.3–14.5
Haematocrit (%)	28.2	33.0–47.0
Neutrophils with band nuclei (%)	16	0–2
Neutrophils with segmented nuclei (%)	71	18–56
Eosinophils (%)	0	1.5–16.1
Basophils (%)	0	0.5–1.4
Monocytes (%)	4	2.7–6.4
Lymphocytes (%)	9	33.9–59.9

### Histological findings

In sow 454 a subacute to acute, moderate to severe, fibrinous pleuropneumonia and pericarditis with pleural and broncho-alveolar exudate consisting of segmented neutrophils, macrophages and fibrin was diagnosed histologically. The pleural, interlobular and peribronchial lymphatic vessels were filled with oedema fluid, neutrophils, macrophages and coccoid bacteria. A severe alveolar and interstitial oedema and a marked hyperaemia of the lung tissue were recorded. The tracheobronchial lymph node was affected by a severe fibrinous-purulent inflammation. A diffuse, proliferative glomerulonephritis was diagnosed as well as a fibrinous serositis on the liver, spleen and uterus serosa surface characterized by neutrophils, fibrin and coccoid bacteria. In the gastric wall coccoid bacteria accompanied by fibrinous clots were found in the lymphatic vessels.

In sow 455 hyperaemia and accumulation of neutrophils in lung and spleen tissue, a glomerulonephritis and an acute, purulent hepatitis were recorded. In ear tissue samples a fibrinous-purulent lymphangitis with oedema fluid, fibrin, neutrophils and focal coccoid bacteria in lymphatic vessels and a perivascular dermatitis were diagnosed.

### Microbiological findings

Microbiological examinations followed the routine standard cultivation protocols in the accredited labs of the Field Station for Epidemiology in Bakum. Selected bacterial colonies were further analysed with an analytical profile index test kit (api 20 Strep ®, Merial, Lyon, France). The api-code 4,463,607 identified *S. zooepidemicus* with an accuracy of 99.9%.

*S. zooepidemicus* was isolated in high yields from sow 454 (> 20 colony forming units (CFU) in a direct cultivation step) from nasal, bronchial, pericardial, thoracal cavity and urinary bladder swabs as well as from lung, spleen and kidney. *S. zooepidemicus* was isolated in low yields (< 10 CFU in a direct cultivation step) from a meningeal swab. The identification of the species was confirmed by MALDI-TOF MS (Matrix-assisted laser desorption time-of-flight) in an external veterinary diagnostic laboratory. The nasal swab and samples from the meningeal swab of sow 455 were also positive for *S. zooepidemicus*. No bacterial pathogens could be isolated from spleen, liver, kidney, lung and thoracal cavity swab of this animal.

*A. pleuropneumoniae*, *Mycoplasma hyopneumoniae*, influenza A virus and PRRSV-EU/-US were not detected by PCR in lung tissue of both animals. Testing for classical swine fever virus, african swine fever virus and porcine herpesvirus 1 by PCR performed in an authorized lab generated also negative results.

Bacteriological culturing of the various organ tissues followed standard procedures for clinical veterinary microbiology [19, 20]. Tissue samples and swabs were plated on four culture plates, as chocolate blood agar containing nicotinamide adenine dinucleotide (NAD, Blood Agar No. 2, Becton, Dickinson and company, Sparks, USA) for culture of *Pasteurellaceae* (e.g. *Glaesserella parasuis*) and *Alcaligenaceae* (e.g. *Bordetella bronchiseptica*), Columbia agar with 5% sheep blood (Becton, Dickinson and company, Sparks, USA), Gassner agar (OXOID, Hampshire, United Kingdom) and CNA blood agar (Becton, Dickinson and company, Sparks, USA) containing polymyxin E and nalidixinic acid for selective culture of *Staphylococcus spp*. and *Streptococcus spp* [21, 22]. Inoculated plates were incubated for 48 h at 37 °C under standard atmospheric conditions, while chocolate blood agar was incubated in an 8% CO_2_ atmosphere. Plates were inspected after 24 and 48 h. For further typing by their cultural and biochemical properties single bacterial colonies were subcultivated. Colonies resembling *A*. *pleuropneumoniae* were subcultivated on chocolate blood agar and tested biochemically for urease, catalase and the CAMP phenomena following routine diagnostic protocols [19].

Antimicrobial susceptibility testing of *S. zooepidemicus* in a microtiter plate assay followed routine diagnostic methods. Interpretation of growth inhibition followed the clinical breakpoints approved by the Clinical and Laboratory Standard Institute [23] and recommended for laboratories in veterinary medicine [24] (Table 2).

**Table 2:** Antibiotic sensitivity test of the isolated *S. zooepidemicus* by MIC (minimal inhibition concentration). S = sensitive, R = resistant

Substance	MIC (µg/ml)	Interpretation
Erythromycin	<=0.12	S
Tulathromycin	<=2	S
Tilmicosin	<=8	S
Tiamulin	<=0.06	S
Penicillin G	<=0.12	S
Ampicillin	<=0.12	S
Amoxycillin/ Clavulanic acid	<=2/1	S
Cephalothin	<=1	S
Ceftiofur	<=0.12	S
Tetracycline	2	S
Trimethoprim/ Sulphonamide	<=0,25/4.75	S
Enrofloxacin	0.25	S
Gentamicin	4	S
Spectinomycin	16	S
Florfenicol	<=1	S
Colistin	> 4	R

### Geno- and phenotyping of the *S. zooepidemicus* isolate

Sequence type (ST) analysis, based on seven highly conserved housekeeping genes (arc, nrdE, proS, spi, tdk, tpi and yqiL) [25], revealed a new sequence type of the *S. zooepidemicus* isolate 21/455, namely 524 [26] (https://pubmlst.org/bigsdb?page=profileInfodb=pubmlst_szooepidemicus_seqdefscheme_id=1profile_id=524; access 20.06.2023). ST524 is a single locus variant to ST65.

**Fig. 5:**
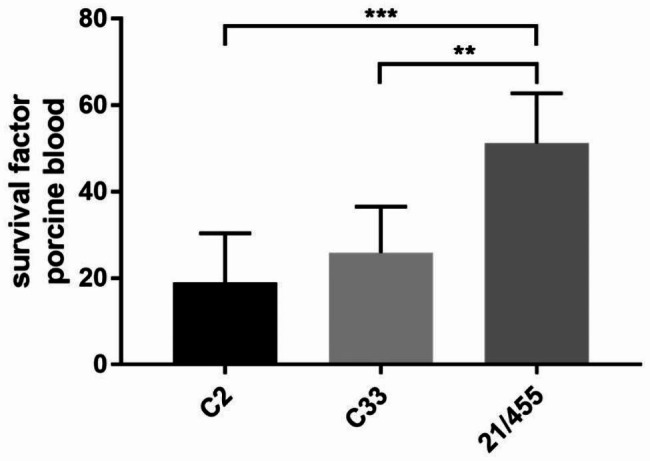
Proliferation of *S. zooepidemicus* 21/455 isolated from a sow of the outbreak described in this case report in comparison to two other *S. zooepidemicus* strains (C2 and C33) characterized in a previous study [16]

At first, the isolate 21/455 showed a shiny, smooth colony phenotype typical for encapsulated strains. This phenotype was, however, lost during further passaging and reappeared after culturing in the presence of porcine serum. We investigated survival of *S. zooepidemicus* strain 21/455 in comparison to two other invasive *S. zooepidemicus* strains [16] in blood of weaning piglets (age: 8 weeks, n = 6) of a herd not affected by this pathogen. The isolate of this outbreak is characterized by a significantly higher proliferation rate in comparison to the other two *S. zooepidemicus* strains (Fig. 5).

### Climate check

A climate check by an agrarian climate expert was focussed onto air flow and ventilation rates in the areas for gestating sows. Approximately 110 sows were kept in groups in one compartment of 10 m width and 26 m length. Three individual exhaust fans at the ceiling with a total power of 12 600 m^3^/h were equipped with gravity valves. The required total summer air exchange rate was calculated by 145 m^3^/h and sow, in total 16 000 m^3^/h and compartment. Temperature sensors were not optimally located at varying positions within the different compartments and ventilation rates were automatically adjusted due to target temperature.

### Case outcome

The final diagnosis based on histopathological and microbiological findings was septicaemia caused by *S. zooepidemicus*. Two weeks after the onset of disease in the farm the veterinarian treated all sows intramusculary with 2 mg cefquinome (Cobactan^®^, MSD Animal Health GmbH, Haar, Germany) per kg body weight on three consecutive days.

No further sows developed fever and sows were no longer apathic. Mild oedemas at the head were still visible in individual sows. In summary, ten sows in late gestation phase died within 4 weeks.

Four weeks after first blood sampling sows were sampled again for paired testing of serum samples in the HIA for influenza A virus antibodies. Antibody titers had decreased, so that an infection with influenza A virus as the causative agent was considered to be unlikely.

Finally, in the short term the change in the antimicrobial treatment with respect to substance and route of administration led to termination of the disease. Neither in this farm nor in other farms in the responsibility of the veterinarian in charge of the stock *S. zooepidemicus* related disease has recurred so far. In the long term in our opinion an important measure on the case farm was a modification of the climate system in the large gestation unit, where the sows had been affected. Additional air supply ducts and air exhaust shafts with larger diameters were installed to increase the air exchange volume while maintaining the same air velocity.

The horse holding site was not modified, although the risk of inter-species transmission was discussed with the farmer.

## Discussion and conclusions

Clinical, histopathological and microbiological findings supported the diagnosis of a sudden sepsis caused by the bacterium *S. equi* sp. *zooepidemicus* in this case. The observed oedema has not been described during *S. zooepidemicus* infection in different species as a specific symptom. It can be assumed that the oedema were a more general sign of septic shock after multi-organ failure due to capillary leaks as a consequence of complement activation and stimulation of granulocytes. The abortion reported for only one sow might be an unspecific finding due to high fever. Nevertheless abortions had been described in horses and also in sows [4, 27].

The first suspicion of the veterinarian – due to the high number of animals affected, high fever, lethargy and anorexia – was an influenza A virus infection. High fever and lethargy in sows are often associated with an influenza A virus infection, but epizootic diseases as Classical or African Swine Fever must be excluded by the respective diagnostic steps. In case of an influenza A virus infection clinical signs in suckling and in nursery piglets would have been expected. Haematological findings were typical for a bacterial infection and the manifestation of inflammation in several organs was typical for a sepsis. Histological findings revealed the presence of coccoid bacteria in inner organs and supported the microbiological findings, so that a septicaemia caused by *S. zooepidemicus* as the causative agent of disease was diagnosed.

*S. zooepidemicus* was also found in meningeal swabs, but unfortunately no histopathological examination was performed. Retrospectively, it would have been of interest, if already histological lesions in meninges and brain tissue were present at the time point of death to assess the date of first exposure. A high bacterial load in the meninges without histological changes has been recorded in early and sudden disease cases before influx of neutrophils takes place. It is known from streptococcal meningitis in human, that bacterial meningitis developed 3–7 days after exposure [28, 29]. After invasion of streptococci throughout the blood-brain barrier the bacterium proliferates at first without being counteracted by any immune mechanisms (no complement and no immunoglobulins present within the subarachnoideal space), so that inflammatory findings can be expected later after the onset of bacterial proliferation [28–30].

No additional disease related-factors as immune-suppression or coinfections with influenza A virus or PRRSV were found. Important differential diagnoses were infections with *M. hyorhinis*, *G. parasuis* and *A. pleuropneumoniae -* all causing serositis in swine and leading to sudden death cases. Due to masses of coccoid bacteria accompanied by an influx of neutrophils within inflammatory tissue alterations *S. zooepidemicus* was assessed as the major pathogen in this case. It cannot be excluded, that other pathogens were also present but had been overgrown by *S. zooepidemicus* in bacteriological culture. So far we have never seen a death case in adult sows due to *M. hyorhinis*, but its involvement in disease pathogenesis as a coinfecting agent can finally not be excluded, as a PCR testing for *M. hyorhinis* was not performed.

An alternative approach for an early diagnosis of septicaemia in sows could be blood cultures, but fever is the only criterium to select appropriate individuals for diagnostic. This means a lack in sensitivity, because in this stage of infection pathogens are already partially eliminated from blood by immune cells. The required aseptical conditions during blood sampling in living pigs are difficult to achieve, so that skin bacteria can distort the findings. In our experience blood culture can be an additional step next to necropsy and bacteriological culture of organ tissue.

Treatment of two sows with doxycycline finally sent for necropsy was not successful, although the isolated *S. zooepidemicus* was assessed as susceptible using the recommended clinical breakpoints: susceptible ≤ 2 mg/L, intermediate: 4 mg/L, resistant ≥ 8 mg/L. In a recent investigation approximately 60% of *S. zooepidemicus* strains from horses were multiresistant to ampicillin, amoxicillin-clavulanate, gentamicin, enrofloxacin, sulfamethoxazole-trimethoprim, tetracycline and other substances [31]. It must be taken into account that for *S. zooepidemicus* in swine no explicit clinical break points exists. If the cut off for tetracycline in *S. suis* (resistant: ≥2) is taken as allocation base, the respective *S. zooepidemicus* strain would have been defined as resistant. Doxycycline was administered orally but diseased sows might had been inappetent, so that no effective concentration of antibacterial substance in infected organ tissue might had been reached. Another reason for treatment failure could be a paradoxon described in human, where in worst scenarios after antibiotic treatment disintegrated cell wall components of died streptococci can trigger a life threatening enhancement of the inflammatory cascade leading to death [28].

An immediate parenteral antibiotic treatment might had been more successful, but was initiated in this case in a second therapeutic approach with cefquinome. Cefquinome is approved for respiratory infections (bronchopneumonia) and has a fast systemic effect. As a fourth generation cephalosporin belonging to the Veterinary Critically Important Antimicrobial Agents cefquinome is not allowed to be used as a first line treatment, but as a second line treatment after susceptibility testing as prescribed by the German Veterinary Pharmacy Regulation. Cefquinome is known to penetrate the blood-brain barrier and is recommended for treatment of septicaemia in swine (OIE List of antimicrobial agents of Veterinary Importance, 2021). It is known to be highly effective against *S. zooepidemicus* infections in horses [32].

Climate check revealed insufficient power of the forced ventilation system under warm summer conditions to guarantee appropriate air exchange rates. In the large gestation unit the exhaust system was error-prone due to gravity valves in the outer part of the exhaust chimney. Wind pressure was causing a back pressure from the outside preventing sufficient opening of the valve. It can be assumed, that real ventilation rates were decreased by this hindrance. Dust particle load in air might had been accumulated under insufficient air exchange rate conditions leading to a high pathogen burden in the environment and in the air. The fact, that only gestating sows were severly affected while sows in other areas recovered after metamizole treatment led to the hypothesis, that insufficient air exchange rates in the large gestational unit triggered the disease.

Gilts were kept in quarantine in a separate building between the horse holding site and the sow farm. Gilts never became ill, so that they were unlikely the source of entry. Finally it cannot be excluded that healthy and immune gilts were carriers of *S. zooepidemicus* leading to introduction of this pathogen to the sow farm. In a recent infection trial faecal shedding, and faecal-oral route were found to be a major route of transmission between pigs. Also healthy carrier pigs were found to shed the pathogen [10].

Another possibility for introduction of the pathogen to the sow farm would be a high bacterial load in the drinking water system with the possibility of an infection by water aerosols during drinking. Unfortunately the authors did not take water samples for examination of *S. zooepidemicus*, so that this route of infection -which is well-known in horses- could not be verified. In the water pipe swabs *S. zooepidemicus* was not detected, but it is not known, if biofilm or water has a higher sensitivity for detection of this pathogen. In the water pipe swabs *staphylococcus spp*. and yeasts were found, which are detected in most water pipe swabs taken in swine husbandry [33]. Water pipes are always covered with biofilm containing also environmental or commensal pathogens. A quantification of microorganisms from water pipe swabs is usually not possible due to the fact, that no measurable amount of water pipe deposit is harvested and that no reference value (e.g. number of pathogens per gram biofilm) exist or can be created. Therefore, swab samples from water pipes are examined mainly for pathogenic microorganisms, which could not be detected in this case. Immunosuppressive or synergistic effects for staphylococci or yeasts are not described, so that an involvement in the disease pathogenesis seems to be unliklely in this case.

Three horses were kept about 100 m away from the sow units. These animals were healthy and were therefore not examined. According to the farmer, the horses have not been ill in the recent past but could have been potential carriers. *S. zooepidemicus* is an opportunistic and commensal pathogen in the upper respiratory tract of horses and can be isolated from clinically healthy carrier animals in high prevalences of about 55% [34–36].

*S. zooepidemicus* is an important swine pathogen in China and South-East Asia. It has also caused outbreaks in pigs in the United States [3, 5]. Interestingly, the outbreak strains from Ohio and Tennessee belong to the same sequence type as two outbreak strains (CY and ATCC 35,246) from China, namely ST194 [7]. The isolate of the case described here is unrelated to ST194. The newly assigned ST524 is a single locus variant of ST65 which includes only five isolates from the upper respiratory tract of horses. This case report shows that this lineage of S. *zooepidemicus* is capable of infecting different hosts such as horses and pigs. In Europe disease in pigs caused by *S. zooepidemicus* has only been described once so far. In this recent case description from the Netherlands identical clinical signs have been reported as high fever, lethargy, mucous nasal discharge, conjunctivitis and swollen eyelids [12]. A sequence type could not be identified.

In conclusion, this case report is the first in Germany describing severe disease in sows caused by an invasive *S. zooepidemicus* infection with a new sequence type.

## Data Availability

Data sharing is not applicable to this article as no datasets were generated or analyzed during the current study.

## Abbreviations

IgG: Immunoglobulin G
PRRSV: Porcine Reproductive and Respiratory Syndrome Virus
PCV2: Porcine Circovirus type 2
HIA: haemagglutination inhibition assay
PCR: polymerase chain reaction
CFU: colony forming units
MALDI: TOF–Matrix–assisted laser desorption time–of–flight
ST: sequence type
